# Postoperative serum oxidative stress measures in women with papillary thyroid cancer

**DOI:** 10.3389/fendo.2026.1810839

**Published:** 2026-04-29

**Authors:** Angelika Buczyńska-Backiel, Iwona Sidorkiewicz, Maria Kościuszko, Katarzyna Siewko, Anna Popławska-Kita, Małgorzata Rusak, Marcin Adamski, Zofia Dzięcioł-Anikiej, Janusz Dzięcioł, Jarosław Szymczuk, Hady Razak Hady, Piotr Myśliwiec, Adam Jacek Krętowski, Agnieszka Adamska

**Affiliations:** 1 Clinical Research Centre, Medical University of Bialystok, Bialystok, Poland; 2 Clinical Research Support Centre, Medical University of Bialystok, Bialystok, Poland; 3 Department of Endocrinology, Diabetology and Internal Medicine, Medical University of Bialystok, Bialystok, Poland; 4 Department of Haematological Diagnostics, Medical University of Bialystok, Bialystok, Poland; 5 Faculty of Computer Science, Bialystok University of Technology, Białystok, Poland; 6 Department of Rehabilitation, Medical University of Bialystok, Bialystok, Poland; 7 Department of Human Anatomy, Medical University of Bialystok, Bialystok, Poland; 8 1st Clinical Department of General and Endocrine Surgery, Medical University of Bialystok, Bialystok, Poland; 9 2nd Clinical Department of General and Gastroenterological Surgery, Medical University of Bialystok, Bialystok, Poland

**Keywords:** 8-oxoguanine DNA glycosylase, angioinvasion, lipid peroxidation, non-invasive biomarkers, oxidative stress, papillary thyroid cancer, ROC analysis

## Abstract

Papillary thyroid carcinoma (PTC) is the most common thyroid malignancy, with a steadily rising incidence worldwide and a marked female predominance. While prognosis is generally favorable, certain histopathological features, particularly angioinvasion, are associated with increased recurrence risk and reduced responsiveness to radioactive iodine (RAI) therapy. Emerging evidence implicates oxidative stress in thyroid tumorigenesis and progression, potentially influencing tumor aggressiveness. Given the higher disease burden in women and the potential influence of hormonal status on oxidative balance, this hypothesis-generating study focused exclusively on female patients to minimize hormonal variability and to explore associations between peripheral oxidative stress measures, DXA-derived body composition parameters, and angioinvasion as a feature of tumor aggressiveness in PTC. A total of 80 women were enrolled, including 36 with angioinvasive PTC, 15 with non-angioinvasive PTC, and 29 healthy controls. Blood samples were collected post-thyroidectomy. A broad panel of biochemical, hormonal, and oxidative stress markers including 3-nitrotyrosine (3-NT), 8-hydroxy-2’-deoxyguanosine (8-OHdG), malondialdehyde (MDA), protein carbonyls, 8-oxoguanine DNA glycosylase-1 (OGG1), and total antioxidant/oxidant capacity (TAS/TAC, TOS) was assessed. Among oxidative stress markers, OGG1 and MDA were significantly elevated in the PTC groups (both, p < 0.001). Additionally, android fat distribution and the android-to-gynoid fat ratio were significantly higher in the angioinvasive group (p = 0.03 and p = 0.01, respectively). Among the evaluated models, the panel combining MDA and TOS demonstrated the highest diagnostic performance (AUC = 0.83), suggesting its potential exploratory value for distinguishing angioinvasive from non-angioinvasive PTC in a postoperative setting.

## 1 Introduction

While the incidence of thyroid cancer (TC) increased markedly over previous decades, more recent data suggest that this trend has stabilized or slightly declined in certain regions, likely reflecting reduced overdiagnosis and more conservative diagnostic practices. Recent reports indicate that incidence rates have decreased by approximately 1–1.5% annually in some populations (1). However, thyroid cancer remains one of the most common endocrine malignancies worldwide, with over 820,000 new cases reported in 2022 (2–5). Papillary thyroid cancer (PTC), accounting for about 90% of TC cases, represents the most rapidly increasing subtype and shows a marked predominance among women (3). Literature data reported a significantly higher TC incidence in females, particularly during reproductive age (6, 7). The ~3:1 female-to-male incidence ratio observed during reproductive years suggests hormonal and reproductive influences, such as early menarche, shorter breastfeeding duration, and premenopausal status, while studies by Tran et al. also implicate sex chromosome–linked molecular and genetic factors. Importantly, several genes involved in oxidative stress response and DNA repair are subject to sex-specific regulation, suggesting that redox balance and oxidative damage processing may differ between women and men (6, 7). Given these observations, sex-specific analyses are essential, as TC exhibits sex chromosome–dependent features, and sex hormones (particularly estrogen and its signaling pathways) may play a pivotal role in the pathogenesis and progression of invasive PTC. In addition, experimental and clinical data suggest that women may exhibit lower baseline oxidative stress levels and higher antioxidant capacity compared to men, partly due to estrogen-mediated upregulation of antioxidant enzymes such as superoxide dismutase and glutathione peroxidase. Conversely, men have been reported to present higher levels of lipid peroxidation and oxidative DNA damage. These sex-specific differences in redox regulation may influence both susceptibility to oxidative damage and tumor behavior in thyroid cancer (6–8). Beyond its proliferative effects, estrogen signaling has been shown to modulate cellular redox homeostasis by influencing mitochondrial activity, reactive oxygen species (ROS) generation, and antioxidant defense mechanisms. Dysregulation of these pathways may enhance oxidative DNA damage and lipid peroxidation, thereby contributing to tumor aggressiveness (8). Beyond hormonal and genetic influences, metabolic factors may also contribute to TC behavior. Increased adiposity, reflected by elevated body mass index (BMI) or an unfavorable fat-to-lean mass ratio, has been associated with more aggressive clinicopathologic features such as extrathyroidal extension, lymph node metastasis, and angioinvasion, although findings remain inconsistent (9–12).

Although PTC generally has a favorable prognosis, recurrence rates vary widely (4–28%) (13, 14). Ito et al. reported nodal, pulmonary, and osseous recurrences in 7%, 2%, and 0.6% of 5,768 patients followed for 10 years, with a 1% disease-specific mortality rate (15). The increasing incidence and recurrence risk of PTC underscore the need to elucidate molecular and histopathological mechanisms of invasiveness, particularly vascular invasion—a key predictor of recurrence and metastasis (16–22). Such insights may enable the development of targeted therapies for improved PTC management. In this context, oxidative stress represents a key biological link between hormonal, genetic, and metabolic influences and tumor aggressiveness in PTC (20–22). Understanding oxidative stress mechanisms may further aid in the development of therapeutic approaches to reduce recurrence and improve clinical outcomes (23, 24). Oxidative stress biomarkers, such as 3-nitrotyrosine (3-NT), 8-hydroxy-2’-deoxyguanosine (8-OHdG), malondialdehyde (MDA), protein carbonyls, and 8-oxoguanine DNA glycosylase-1 (OGG1) serve as critical indicators for assessing cellular and molecular damage induced by ROS. The assessment of these biomarkers offers mechanistic insights into oxidative stress–related disorders and holds potential clinical utility in disease monitoring, prognosis, and therapeutic decision-making (16, 25).

Focusing exclusively on female patients allowed us to reduce biological variability related to sex-specific hormonal and genetic factors and to investigate oxidative stress markers within a more homogeneous redox and metabolic background. It integrates the assessment of peripheral oxidative stress biomarkers with detailed body composition analysis using dual-energy X-ray absorptiometry (DXA) to provide a comprehensive evaluation of systemic redox balance and metabolic profile. The primary objective of this study was to evaluate whether systemic oxidative stress biomarkers can discriminate between angioinvasive and non-angioinvasive PTC in a postoperative setting. Secondary analyses included comparisons of oxidative stress and metabolic parameters across all three groups (angioinvasive PTC, non-angioinvasive PTC, and healthy controls) to provide broader biological context.

## 2 Materials and methods

### 2.1 Study population

This study was conducted at the Department of Endocrinology, Diabetology, and Internal Medicine, Medical University of Bialystok. The study included women aged 18 to 65 years. All patients with PTC were diagnosed based on pathomorphological examination. A total of 200 patients with varying stages of PTC following total thyroidectomy, who were referred to the Department of Endocrinology, Diabetology, and Internal Medicine for qualification for radioiodine treatment (RAI), were screened. Of the initial cohort of 200 consecutive PTC patients (both sexes), only female patients were included in the present analysis to reduce sex-related biological variability; the final study groups were defined after applying this criterion together with predefined inclusion and exclusion criteria. All participants provided written informed consent, and the study was conducted in accordance with ethical standards.

The study was conducted in accordance with the Declaration of Helsinki and approved by the Ethics Committee of the Medical University of Bialystok, Poland (approval no. APK.002.66.2025). All diagnostic procedures were in accordance with the current recommendations of the Polish Endocrine Society (PTE) from 2022, the American Thyroid Association (ATA) from 2025, and the National Comprehensive Cancer Network (NCCN) (26, 27).

After applying sex-specific with age selection and exclusion criteria, 51 female patients with complete datasets were eligible for the final analysis. To assess the clinical utility of selected serum parameters as potential indicators of angioinvasion, the study group consisted of patients with PTC showing features of angioinvasion (targeting 36 patients), while the reference group included patients with PTC without these features (reference group – targeting 15 patients). Additionally, a control group consisting of 29 healthy women who voluntarily agreed to participate in the study was recruited. The control group comprised apparently healthy women aged 18–65 years with no history or clinical evidence of thyroid disease. Normal thyroid status in the control group was confirmed by biochemical assessment (TSH, fT3, and fT4), which was available for all participants and within the reference range. Thyroid ultrasound was additionally performed in a subset of participants when available to confirmed control group enrolment. Thus, healthy controls were carefully matched to the study and reference groups, selected under strict exclusion criteria, and evaluated using comprehensive DXA-derived body composition measures to minimize confounding related to metabolic status. Given that subclinical thyroid dysfunction may be asymptomatic, particularly in postmenopausal women, strict exclusion criteria and biochemical evaluation were applied to minimize this risk.

### 2.2 Exclusion criteria

Individuals were excluded if they met any of the following conditions: (1) history of other malignancies or recurrent thyroid cancer; (2) presence of autoimmune disorders, including active or clinically significant systemic autoimmune conditions (e.g., rheumatoid arthritis, systemic lupus erythematosus); (3) chronic inflammatory or infectious diseases; (4) metabolic disorders such as uncontrolled diabetes mellitus or dyslipidemia requiring pharmacological treatment; (5) liver or kidney dysfunction (ALT or AST >3× upper limit of normal (ULN), eGFR <60 mL/min/1.73 m²); (6) cardiovascular disease within the past 6 months; (7) current use of antioxidant supplementation, hormone replacement therapy, or corticosteroids; (8) smoking or excessive alcohol consumption; (9) pregnancy or lactation; (10) age <18 or >65 years.

### 2.3 Study scheme

Blood samples were collected under standardized conditions from the antecubital vein, allowed to clot at room temperature, and centrifuged to obtain serum. Samples were aliquoted and stored at −80 °C until analysis. All samples were processed within a consistent time frame and analyzed in the same batch to minimize pre-analytical variability.

At the time of blood collection (3–5 months after total thyroidectomy), all PTC patients were receiving levothyroxine therapy individually adjusted to achieve TSH suppression according to current clinical guidelines. The achieved thyroid status is reflected by measured TSH, fT4, and fT3 concentrations at the time of sampling (**Table 1**). Blood samples were collected before the qualification phase for RAI therapy; therefore, none of the patients had received RAI prior to sampling. Patients with acute infections, or inflammatory conditions were excluded based on predefined criteria. Additionally, CRP levels were within a low range across study groups, supporting the absence of active inflammatory processes at the time of sampling.

**Table 1:** Biochemical profiling of studied groups (*the Kruskal–Wallis test followed by Dunn’s *post hoc* test with correction for multiple comparisons).

Parameter	Units	Control group	PTC group		*p*-Value*
			*without angioinvasion (reference group)*	with angioinvasion (study group)	
WBC	(*10^3/µI)	5.1 (4.55-6.1)	6.6 (4.8-7.3)	6.51 (5.31-8.96)	>0.05
Glucose	(mg/dL)	90.0 (85.5-94.0)	90.0 (85.0-96.0)	97.5 (92-106.5)	>0.05
CREAT	(mg/dl)	0.73 (0.7-0.8)	0.7 (0.6-0.7)	0.68 (0.6-0.7)	0.006
CHOL	(mg/dL)	166 (154.5–181.5)	223.0 (212.0–241.0)	195.5 (173.3–230.3)	<0.0001
HDL	(mg/dL)	76.0 (70.4–86.7)	67.0 (60.0–74.0)	55.0 (48.25–65.0)	0.02
LDL	(mg/dL)	80.6 (67.0–93.5)	128.0 (119.0–168.0)	121.5 (92.5–142.8)	<0.0001
AST	(U/L)	17.4 (16.0-21.6)	23.0 (19.0-28.0)	21.5 (20.0-24.0)	0.001
ALT	(U/L)	12.2 (9.8-16.5)	18.0 (15.0-28.0)	23.0 (16.3-29.8)	0.001
CRP	(mg/L)	0.82 (0.5–1.6)	1.3 (1.0–1.7)	1.9 (1.0–5.025)	0.02
TSH	(µIU/mL)	1.9 (1.49–2.8)	0.5 (0.1–2.9)	0.4 (0.1–1.8)	0.009
fT3	(pg/mL)	3.2 (2.7–3.6)	2.8 (2.5–3.0)	2.7 (2.4–3.1)	0.004
fT4	(ng/dL)	1.2 (1.1–1.3)	1.1 (1.0–1.2)	1.1 (1.0–1.2)	0.04
TgAb	(IU/mL)	0.5 (0.4- 1.9)	1.8 (0.7-4.2)	3.1 (1.2–6.9)	0.041
3-NT	(ng/ml)	23.9 (19.3-29.6)	28.1 (20.3-31.5)	18.4 (14.2-26.7)	>0.05
OGG-1	(ng/ml)	5.1 (4.8-6.1)	6.6 (6.1-8.3)	6.4 (5.6-6.9)	0.0003
8OHdG	(pg/ml)	887.3 (707.4-1426.0)	1119.0 (835.6-1995.0)	1020.0 (798.0-1453.0)	>0.05
TOS	(pg/ml)	12.5 (8.1-65.0)	72.2 (14.5-160.9)	12.3 (6.3-54.9)	>0.05
MDA	(µmol)	2.7 (2.1-3.3)	18.4 (17.2-25.6)	6.1 (2.4-16.6)	<0.0001
Protein Carbonyl	(nmol/ml)	247.5 (192.8-326.7)	200.7 (149.3-253.6)	211.6 (138.4-322.1)	>0.05
TAS/TAC	(µmol/l)	323 (279.9-359.7)	294.6 (219.4-371.1)	347.5 (290.0-377.1)	>0.05
TISSUE*	(% FAT)	33 (28.6–38.9)	36.8 (33.1–42.6)	43.05 (36.6–47.3)	>0.05
TOTAL MASS*	(kg)	64.6 (56.8–73.8)	69 (55.9–75.8)	76.5 (66.7–94.2)	>0.05
REGION*	(% FAT)	31.7 (27.6–37.4)	35.3 (31.8–41.3)	41.9 (35.3–45.9)	>0.05
ANDROID*	(% FAT)	30.6 (21.6-40.4)	37.4 (33.8-48.6)	48.8 (34.7-52.5)	0.03
GYNOID*	(% FAT)	38.4 (34.9-42.9)	41 (36.3-46.6)	44.15 (38.5-48.1)	>0.05
A/G RATIO*		0.8 (0.6-1.0)	1.0 (0.86-1.1)	1.055 (0.94-1.1)	0.01
TOTAL BODY*	(% FAT)	33 (28.6-38.9)	36.8 (33.1-42.6)	43.1 (36.55-47.3)	>0.05
BMI		21.9 (20.3-24.4)	23.9 (21.5-27.8)	28.9 (25.1-34.7)	>0.05

WBC, white blood cell count; Glucose, blood glucose concentration; CREAT, creatinine; CHOL, total cholesterol; HDL, high-density lipoprotein; LDL, low-density lipoprotein; AST, aspartate aminotransferase; ALT, alanine aminotransferase; CRP, C-reactive protein; TSH, thyroid-stimulating hormone; fT3, free triiodothyronine; fT4, free thyroxine; TgAb, thyroglobulin antibodies; 3-NT, 3-nitrotyrosine; OGG1, 8-oxoguanine DNA glycosylase-1; 8OHdG, 8-hydroxy-2’-deoxyguanosine; TOS, total oxidant status; MDA, thiobarbituric acid reactive substances; Protein Carbonyl, protein carbonyl content; TAS/TAC, total antioxidant status/capacity; TISSUE, total fat tissue; TOTAL MASS, body weight; REGION, regional fat; FAT, fat mass; LEAN, lean body mass; FAT FREE, fat-free mass; ANDROID, android fat; GYNOID, gynoid fat; A/G RATIO, android-to-gynoid fat ratio; TOTAL BODY, total body fat; BMI, body mass index.

Thus, blood samples were collected during the postoperative follow-up visit, approximately 3–5 months after total thyroidectomy, once the final histopathological diagnosis was available. This timing was consistent across both PTC groups, and no samples were obtained in the immediate postoperative period. The presence of angioinvasion was assessed postoperatively based on histopathological examination of surgical specimens, according to established pathological criteria. Histopathological evaluation was performed by experienced pathologists in accordance with standard diagnostic criteria (28–30). A single blood sample was collected from participants in all groups, in the same manner as for the study and reference groups. All samples were frozen and stored at -80 °C. All participants underwent DXA using Lunar iDXA (GE Healthcare, USA). The total amount of lean body mass (LBM), fat mass (FM) and visceral adipose tissue mass (VAT mass) were measured. In healthy controls, absence of thyroid disease was further supported by normal thyroid ultrasound findings when available and by the lack of clinical or biochemical features suggestive of thyroid dysfunction.

### 2.4 Biochemical assessment

Blood samples were collected from the antecubital fossa, prepared for biochemical analyses, and stored at −80 °C for future research.

The concentrations of thyroid-stimulating hormone (TSH), free triiodothyronine (fT3), free thyroxine (fT4), thyroglobulin (Tg), and thyroglobulin antibodies (TgAb) were measured using the electrochemiluminescence immunoassay (ECLIA) on the Roche E411 analyzer (Roche Diagnostics, Sussex, UK).

Biochemical parameters including triglycerides (TG), total cholesterol (CHOL), high-density lipoprotein (HDL), low-density lipoprotein (LDL), glucose, C-reactive protein (CRP), and creatinine (CREAT) were determined using the enzymatic colorimetric method on the Roche C111 analyzer (Roche Diagnostics, Basel, Switzerland).

White blood cell (WBC) count was assessed as part of the complete blood count using the Mythic 18 automated hematology analyzer (Orphée, Geneva, Switzerland).

Total oxidant status (TOS) was measured using a photometric immunodiagnostic assay (PerOx (TOS/TOC) Kit, KC5100, Bensheim, Germany), and total antioxidant status/capacity (TAS/TAC) was assessed using the ImAnOx (TAS/TAC) Kit (KC5200, Bensheim, Germany), both according to the manufacturer’s instructions. Oxidative stress markers, including 3-NT, OGG1, and 8-OHdG were determined using ELISA kits (Cloud Clone Corp., Wuhan, China; product codes CEB863Ge, SEC704Hu, and CEA660Ge, respectively). MDA concentration, as a measure of lipid peroxidation, was quantified using the thiobarbituric acid reactive substances (MDA) assay (Cayman Chemical, USA; Cat. No. 10009055) following the manufacturer’s protocol. Protein oxidation was assessed by measuring protein carbonyl content using the Protein Carbonyl Colorimetric Assay Kit (Cayman Chemical, USA; Cat. No. 10005020). All assays were performed according to the manufacturers’ protocols, including recommended calibration procedures and quality control measures. Analytical performance characteristics, such as detection limits and intra- and inter-assay variability, were within acceptable ranges as specified by the manufacturers.

Body composition parameters, including total body mass, fat mass (FAT), lean body mass (LEAN), fat-free mass (FAT FREE), android and gynoid fat distribution (ANDROID, GYNOID), total fat percentage total body, tissue, and android-to-gynoid fat ratio (A/G RATIO) were assessed using dual-energy X-ray absorptiometry (DXA) with a Lunar iDXA scanner (GE Healthcare, USA).Samples and controls were measured using the blind analysis method in the same run.

### 2.5 Statistical analysis

All analyses were conducted using blinded procedures to minimize potential bias. Descriptive statistics included mean, standard deviation, median, minimum and maximum values, as well as 95% confidence intervals. The Shapiro–Wilk test was used to assess the normality of data distribution. As the assumption of normality was not met, non-parametric methods were applied. Comparisons among the three groups (controls, non-angioinvasive PTC, and angioinvasive PTC) were performed using the Kruskal–Wallis test. When the overall test was significant, *post hoc* pairwise comparisons were conducted using Dunn’s test with adjustment for multiple comparisons, as implemented in GraphPad Prism. Correlations between variables were assessed using Spearman’s rank correlation coefficient. Receiver Operating Characteristic (ROC) curves were generated to evaluate the diagnostic performance of individual biomarkers and their combinations in distinguishing between angioinvasive and non-angioinvasive PTC. The area under the curve (AUC) with 95% confidence intervals was calculated.

To assess the combined diagnostic performance of selected biomarkers, multivariable logistic regression models were constructed with angioinvasion status (yes/no) as the dependent variable. Candidate variables were selected based on biological relevance and prior analyses, with a focus on oxidative stress markers. No automated or data-driven variable selection procedures were applied. Given the limited sample size, the number of predictors included in each model was restricted to reduce the risk of overfitting; therefore, primarily two-marker combinations were evaluated. The predicted probabilities derived from the models were used to generate ROC curves. For multivariable models, a classification threshold of 0.5 was applied. Sensitivity and specificity at the selected thresholds were calculated. Due to the exploratory nature of the study and limited sample size, positive and negative predictive values were not emphasized. No external validation was performed; therefore, the results of multivariable models should be interpreted as exploratory.

Statistical significance was set at p < 0.05. All analyses were performed using GraphPad Prism 10 (GraphPad Software, San Diego, CA, USA). The primary analysis focused on differences between angioinvasive and non-angioinvasive PTC patients. Comparisons including the control group and correlation analyses were considered secondary and exploratory. Similarly, multivariable models and biomarker panels were evaluated in an exploratory manner. Due to the limited sample size and the risk of model overfitting, multivariable models were not adjusted for multiple clinical confounders. Instead, a parsimonious modeling strategy was applied, restricting the number of predictors included in each model. Potential confounding was partially addressed through strict inclusion and exclusion criteria and by ensuring relative homogeneity of the study groups. Variables reflecting key confounding domains, including age, adiposity (DXA-derived parameters), inflammatory marker (CRP), lipid profile, and thyroid function tests, were analyzed separately to provide clinical context.

## 3 Results

In accordance with the predefined analysis hierarchy, primary analyses focused on comparisons between angioinvasive and non-angioinvasive PTC patients, while comparisons including the control group were considered exploratory. Key clinical and metabolic parameters potentially acting as confounders, including adiposity measures, lipid profile, inflammatory marker, and thyroid function tests, are presented in **Table 1** to provide context for the observed associations (**Table 1**).

### 3.1 Basic biochemical parameters

No significant differences were observed in WBC count or glucose levels between the PTC groups (with and without angioinvasion) and the control group (p > 0.05). However, CREAT levels were significantly lower in the angioinvasive PTC group compared to controls (p = 0.006).

Patients with PTC, particularly those with angioinvasion, demonstrated significant dyslipidemia. CHOL and LDL concentrations were markedly elevated in both PTC groups in comparison to the control group (p < 0.0001). Conversely, HDL levels were significantly reduced in the angioinvasive group relative to controls (p = 0.02).

Liver enzymes, AST and ALT were significantly higher in both PTC groups compared to the control group (p = 0.001, all). Additionally, CRP levels were significantly elevated in the angioinvasive PTC group compared to controls (p = 0.02) (**Table 1**).

### 3.2 Thyroid function and autoimmunity

TSH levels were significantly decreased in the angioinvasive PTC group compared to the control group (p = 0.009). Both fT3 and fT4 concentrations were also reduced in PTC patients, regardless of angioinvasion status, compared to controls (p = 0.004 and p = 0.04, respectively). Additionally, a significant increase in TgAb levels was observed in the angioinvasive group compared to controls (p = 0.041) (**Table 1**). These differences in thyroid hormone parameters should be interpreted in the context of postoperative management. All PTC patients were receiving levothyroxine therapy aimed at TSH suppression according to current clinical guidelines. Therefore, the significantly lower TSH levels observed in PTC patients compared to controls reflect the expected effect of treatment rather than disease-specific alterations. Similarly, variability in fT3 and fT4 concentrations may be related to individual differences in hormone replacement therapy and peripheral thyroid hormone metabolism. Thus, these findings primarily represent treatment-related physiology rather than tumor-driven effects.

### 3.3 Oxidative stress biomarkers

**Figure 1:**
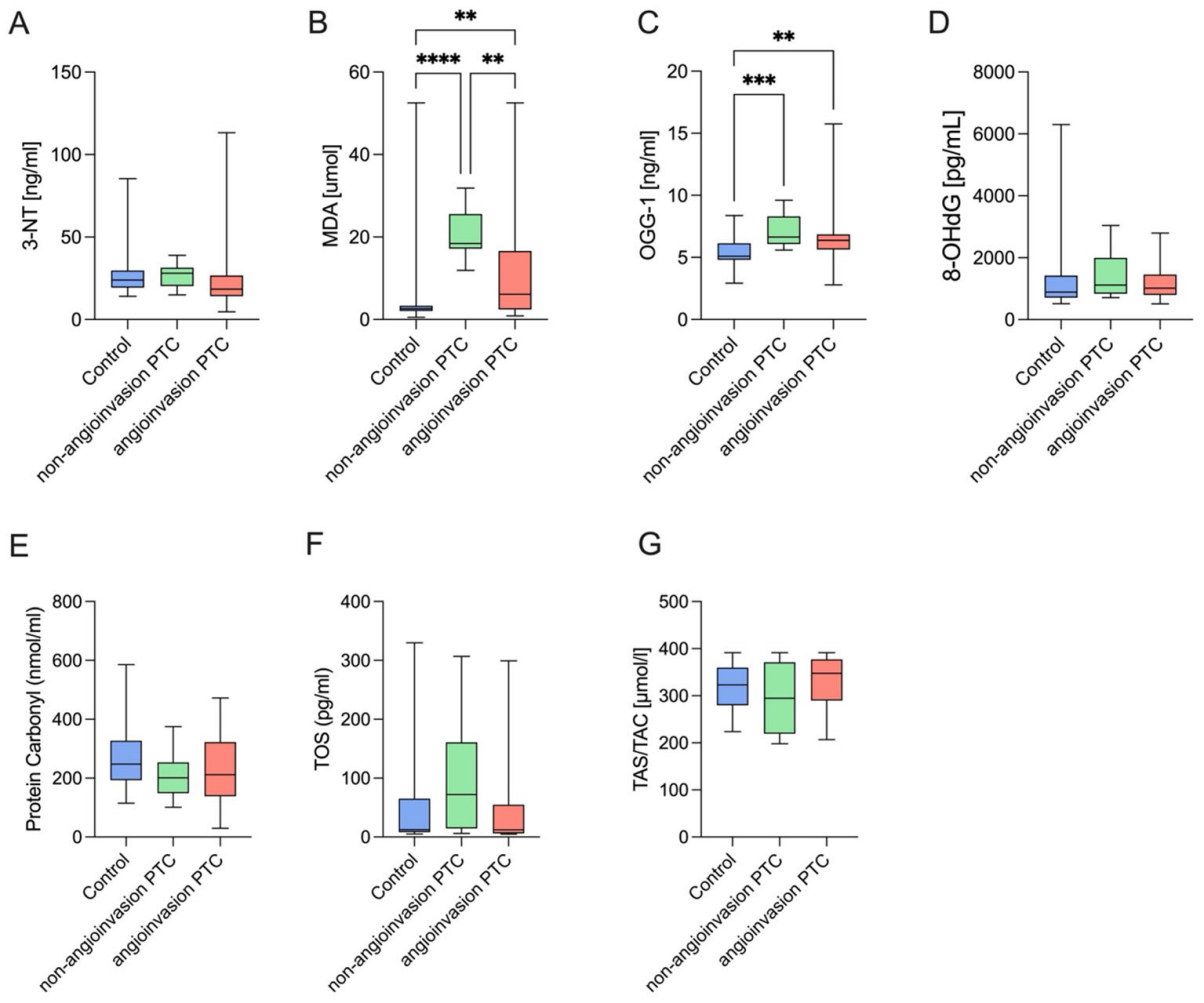
Comparison of oxidative stress markers among study groups. Box plots illustrate serum concentrations of selected oxidative stress biomarkers in the control group, non-angioinvasive PTC group, and angioinvasive PTC group; **(A)** 3-nitrotyrosine (3-NT), **(B)** thiobarbituric acid reactive substances (MDA), **(C)** 8-oxoguanine DNA glycosylase-1 (OGG1), **(D)** 8-hydroxy-2’-deoxyguanosine (8-OHdG), **(E)** protein carbonyl content, **(F)** total oxidant status (TOS), **(G)** total antioxidant status/capacity (TAS/TAC). (Data are presented as medians with interquartile ranges including individual data points overlaid on box plots. Statistical significance was assessed using the Kruskal–Wallis test followed by Dunn’s *post hoc* test with correction for multiple comparisons (*p < 0.05, **p < 0.01, ***p < 0.001, ****p < 0.0001)).

Among oxidative stress markers, OGG1 concentrations were significantly elevated in both PTC groups compared to the control group (p = 0.0003), indicating increased oxidative DNA damage repair activity. MDA levels, reflecting lipid peroxidation, were markedly higher in the PTC group without angioinvasion and moderately elevated in the angioinvasive group, relative to controls (p < 0.0001). In contrast, no significant differences were observed in 3-NT, 8OHdG, TOS, protein carbonyls, or TAS/TAC across the studied groups (p > 0.05) (**Table 1**, **Figure 1**).

### 3.4 Body composition

Although no significant differences were observed in most body composition parameters including BMI, FAT, or LEAN, both ANDROID fat distribution and the A/G RATIO were significantly higher in the angioinvasive PTC group compared to controls (p = 0.03 and p = 0.01, respectively) (**Table 1**).

### 3.5 Correlation matrix

In the control group, several significant correlations were observed between oxidative stress markers and metabolic, hormonal, or inflammatory parameters. 3-NT levels showed a negative correlation with fT3 (r = –0.42, p = 0.02), while OGG1 positively correlated with fT4 (r = 0.40, p = 0.03). TOS correlated positively with both TSH (r = 0.39, p = 0.04) and age (r = 0.43, p = 0.02). MDA levels showed strong positive correlations with CHOL (r = 0.52, p = 0.005), HDL (r = 0.61, p = 0.0006), and CRP (r = 0.39, p = 0.04). In turn, protein carbonyls were inversely associated with RBC (r = –0.44, p = 0.02) and A/G RATIO (r = –0.45, p = 0.01). The TAS/TAC index positively correlated with vitamin D (r = 0.52, p = 0.004) and negatively with TOS (r = –0.45, p = 0.01).

In reference group, OGG1 levels positively correlated with WBC (r = 0.51, p = 0.049) and RBC (r = 0.68, p = 0.005). 8OHdG was positively associated with TSH (r = 0.52, p = 0.045) and negatively with fT4 (r = –0.54, p = 0.04). Both TOS and MDA correlated positively with CHOL and CRP; additionally, MDA was strongly associated with LDL (r = 0.74, p = 0.002). Notably, TAS/TAC showed an inverse correlation with CHOL (r = –0.64, p = 0.01) and a positive one with vitamin D (r = 0.59, p = 0.02).

In patients with angioinvasive PTC, a broader network of significant correlations was observed. 3-NT positively correlated with TgAb (r = 0.38, p = 0.02), while OGG1 showed an inverse association with Tg (r = –0.47, p = 0.04). 8OHdG negatively correlated with multiple parameters reflecting body composition, including TISSUE (%FAT), TOTAL MASS, REGION (%FAT), ANDROID (%FAT), GYNOID (%FAT), BMI, and A/G RATIO (r range = –0.34 to –0.49, p < 0.05). TOS positively correlated with CHOL, HDL, and LDL (r =0.47–0.49, p < 0.005), while showing negative correlations with CRP, fT4, and PLT. MDA was significantly associated with CHOL, inversely with fT3, and strongly negatively correlated with TgAb (r = –0.53, p = 0.001). Protein carbonyls demonstrated positive associations with HDL and fT3, and a negative correlation with TSH (r = –0.47, p = 0.004).

The results of the correlation analysis are presented in **Table 2**.

**Table 2:** Correlation matrix (*only significant associations-assessed using Spearman’s rank correlation coefficient).

Correlations between investigated parameters in control group			
correlation coefficient (r) *p-value			
3-NT	fT3	-0.42	0.02
OGG1	fT4	0.4	0.03
TOS	TSH	0.39	0.04
	AGE	0.43	0.02
MDA	CHOL	0.52	0.005
	HDL	0.61	0.0006
	CRP	0.39	0.04
Protein Carbonyl	RBC	-0.44	0.02
	A/G RATIO*	-0.45	0.01
TAS/TAC	WIT D	0.52	0.004
	TOS	-0.45	0.01
Correlations between investigated parameters in PTC group without angioinvasion (reference group)			
OGG1	WBC	0.51	0.049
	RBC	0.68	0.005
8OHdG	TSH	0.52	0.045
	fT4	-0.54	0.04
TOS	CHOL	0.66	0.007
	CRP	0.58	0.02
MDA	CHOL	0.59	0.02
	LDL	0.74	0.002
TAS/TAC	CHOL	-0.64	0.01
	WIT D	0.59	0.02
Correlations between investigated parameters in PTC group with angioinvasion (study group)			
3-NT	TgAb	0.38	0.02
OGG1	Tg	-0.47	0.04
8OHdG	WBC	-0.45	0.006
	TISSUE (%FAT)*	-0.42	0.01
	CENTILE*	-0.49	0.003
	TOTAL MASS*	-0.43	0.01
	REGION (%FAT)*	-0.42	0.01
	ANDROID (%FAT)*	-0.38	0.02
	GYNOID (%FAT)*	-0.41	0.02
	A/G RATIO*	-0.35	0.04
	TOTAL BODY (%FAT)*	-0.42	0.01
	BMI	-0.44	0.009
TOS	CHOL	0.49	0.002
	HDL	0.47	0.003
	LDL	0.49	0.003
	CRP	-0.34	0.04
	fT4	-0.43	0.009
	PLT	-0.44	0.007
MDA	CHOL	0.33	0.046
	fT3	-0.35	0.04
	TgAB	-0.53	0.001
Protein Carbonyl	HDL	0.4	0.02
	TSH	-0.47	0.004
	fT3	0.45	0.006

WBC, white blood cell count; Glucose, blood glucose concentration; CREAT, creatinine; CHOL, total cholesterol; HDL, high-density lipoprotein; LDL, low-density lipoprotein; AST, aspartate aminotransferase; ALT, alanine aminotransferase; CRP, C-reactive protein; TSH, thyroid-stimulating hormone; fT3, free triiodothyronine; fT4, free thyroxine; TgAb, thyroglobulin antibodies; 3-NT, 3-nitrotyrosine; OGG1, 8-oxoguanine DNA glycosylase-1; 8OHdG, 8-hydroxy-2’-deoxyguanosine; TOS, total oxidant status; MDA, thiobarbituric acid reactive substances; Protein Carbonyl, protein carbonyl content; TAS/TAC, total antioxidant status/capacity; TISSUE, total fat tissue; TOTAL MASS, body weight; REGION, regional fat; TISSUE, total tissue mass; FAT, fat mass; LEAN, lean body mass; FAT FREE, fat-free mass; ANDROID, android fat; GYNOID, gynoid fat; A/G RATIO, android-to-gynoid fat ratio; TOTAL BODY, total body fat; BMI, body mass index; *based on DXA.

### 3.6 Exploratory evaluation of oxidative stress marker panels

The area under the ROC curve (AUC) for OGG1 was 0.7213 (p < 0.001), while MDA yielded a comparable AUC of 0.7300 (p = 0.001), indicating similar discriminative ability of both markers in predicting angioinvasion in PTC.

**Figure 2:**
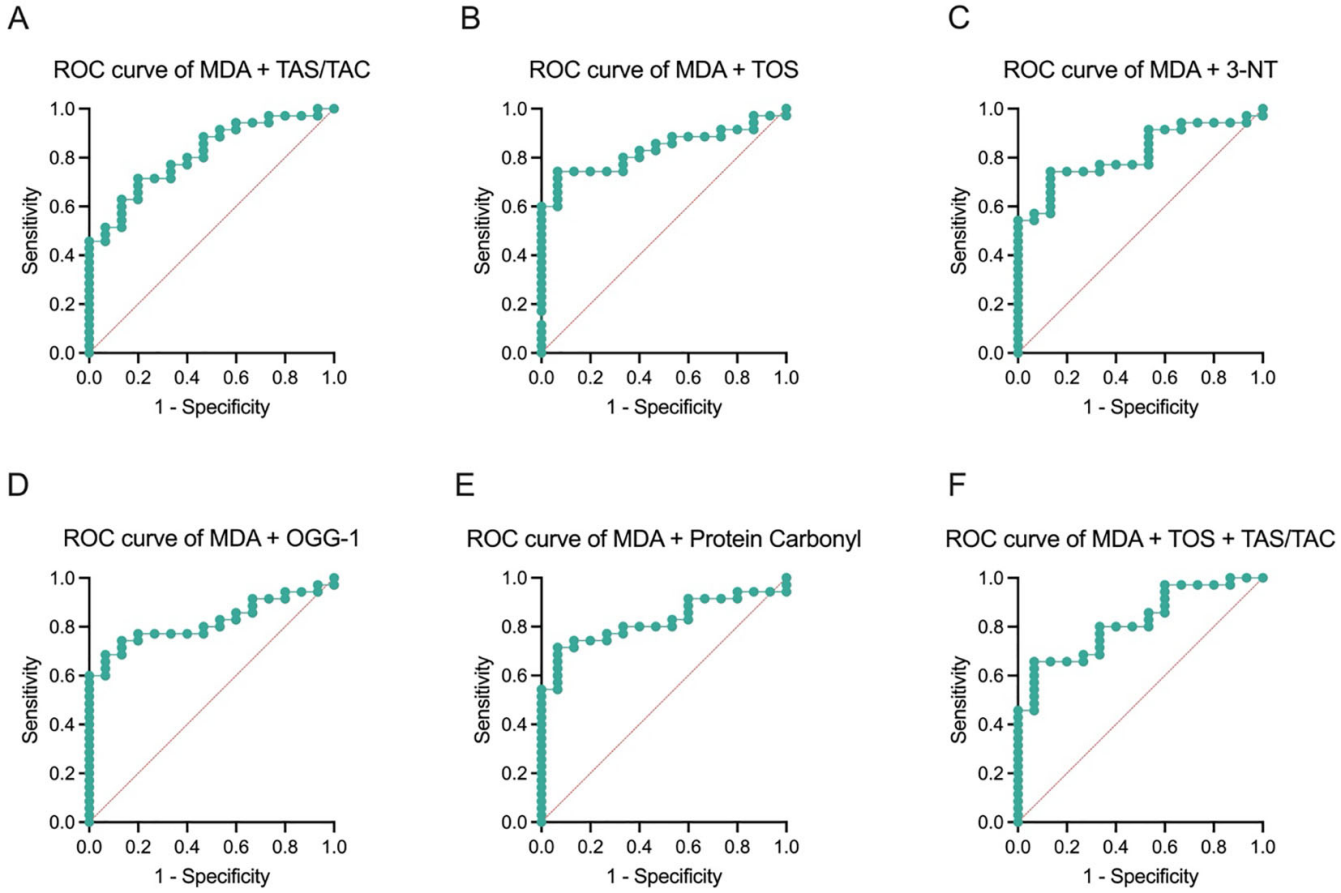
Diagnostic performance of selected oxidative stress markers for the identification of angioinvasion in PTC patients (**(A)** MDA+TAS/TAC; **(B)** MDA + TOS; **(C)** MDA + 3-NT; **(D)** MDA + OGG-1; **(E)** MDA + Protein Carbonyl, **(F)** MDA + TOS + TAS/TAC).

Given the moderate diagnostic performance of individual oxidative stress markers, a multivariate approach was undertaken to enhance discriminative capacity for angioinvasive PTC. For this purpose, several diagnostic panels combining oxidative, inflammatory, and lipid-related variables were constructed and evaluated using logistic regression and ROC curve analysis. All models incorporated MDA as a common oxidative stress component due to its consistent association with angioinvasion across analyses. Each panel included an additional marker, such as TAS/TAC, TOS, 3-NT, OGG1, or protein carbonyls, depending on the biological pathway targeted. Notably, the panel combining MDA and TAS/TAC demonstrated high discriminative power with an AUC of 0.8133 (95% CI: 0.6937–0.9330; p = 0.0005). Similarly, the TOS + MDA model yielded an AUC of 0.8324 (95% CI: 0.7221–0.9426; p = 0.0002), and the 3-NT + MDA panel reached an AUC of 0.8152 (95% CI: 0.6987–0.9318; p = 0.0005). Other combinations, such as OGG1 + MDA and Protein Carbonyls + MDA, also showed robust performance with AUC values ranging from 0.8190 to 0.8220, all with statistically significant p-values (p ≤ 0.0004). The most complex model, integrating TOS, MDA, and TAS/TAC, achieved an AUC of 0.8171 (95% CI: 0.6997–0.9346; p = 0.0004) for angioinvasion classification in PTC patients, suggesting that multi-marker approaches may provide additional diagnostic value over single parameters. Among the evaluated models, the panel combining MDA and TOS showed moderate to high discriminative performance (AUC = 0.8324), indicating its potential utility as a simple and biologically informative marker combination in this exploratory cohort, which should be interpreted cautiously due to limited sample size (**Figure 2**, **Table 3**). Across all two-marker models, high sensitivity was consistently observed, ranging from 88.6% to 94.3%, whereas specificity remained relatively low (26.7–40.0%). Positive predictive values ranged from 74.4% to 78.1%, while negative predictive values ranged from 55.6% to 71.4%. Notably, several models, including OGG1 + MDA and protein carbonyls + MDA, demonstrated identical classification performance, indicating overlapping predictive information among oxidative stress markers. The three-marker model (TOS + MDA + TAS/TAC) showed a more balanced performance profile, with sensitivity of 85.7% and improved specificity of 46.7%, compared to two-marker panels, while maintaining comparable predictive values (PPV 78.9%, NPV 58.3%). Overall, these findings indicate that while two-marker panels provide high sensitivity, the addition of a third biomarker may modestly improve specificity, suggesting a trade-off between model simplicity and classification balance.

**Table 3:** Summary of the basic parameters and common quality measures of the models (cut off = 0.5).

Model	Odds ratios	Variable	Estimate	95% CI (profile likelihood)	AUC	SE	95% CI	P value	Sensitivity/Specificity	PPV/NPV
MDA + TAS/TAC	ß0	Intercept	0.23	0.00 - 12.24	0.81	0.06	0.69 – 0.93	0.0005	91.40%/40.00%	78.05%/66.67%
	ß1	MDA [umol]	0.91	0.82 – 0.97						
	ß2	TAS/TAC [umol/l]	1.01	1.00 – 1.03						
MDA + TOS	ß0	Intercept	15.01	3.78 – 90.08	0.83	0.06	0.7221 – 0.9426	0.0002	88.60%/33.30%	75.61%/55.56%
	ß1	MDA [umol]	0.91	0.83 – 0.97						
	ß2	TOS [pg/l]	1.00	0.99 – 1.00						
MDA + 3-NT	ß0	Intercept	24.81	3.73 – 293.90	0.82	0.06	0.70 – 0.93	0.0005	94.30%/33.30%	76.74%/71.43%
	ß1	MDA [umol]	0.90	0.81 – 0.97						
	ß2	3-NT [ng/ml]	0.98	0.93 – 1.02						
MDA + OGG-1	ß0	Intercept	23.23	1.43– 520.00	0.82	0.06	0.71 – 0.93	0.0004	91.40%/26.70%	74.42%/57.14%
	ß1	MDA [umol]	0.90	0.83 – 0.96						
	ß2	OGG-1 [ng/ml]	0.90	0.65 – 1.32						
MDA + Protein Carbonyl	ß0	Intercept	6.75	0.90 – 68.54	0.82	0.06	0.71 – 0.94	0.004	91.40%/26.70%	74.42%/57.14%
	ß1	MDA [umol]	0.90	0.83 – 0.97						
	ß2	Protein Carbonyl [nmol/ml]	1.00	1.00 – 1.01						
MDA+TOS+TAS/TAC	ß0	Intercept	0.30	0.00 – 18.53	0.82	0.06	0.70 – 0.94	0.0004	85.70%/46.70%	78.90%/58.30%
	ß1	MDA [umol]	0.91	0.83 – 0.97						
	ß2	TOS [pg/l]	1.0	0.99 – 1.00						
	ß3	TAS/TAC [umol/l]	1.01	1.00 – 1.03						

## 4 Discussion

This study shows that selected oxidative stress markers, particularly MDA and OGG1, differ between PTC patients and controls and are associated with angioinvasion. Multi-marker panels demonstrated moderate discriminative performance, with high sensitivity but limited specificity. Across the evaluated models, high sensitivity (approximately 85–94%) was consistently observed, whereas specificity remained relatively low (approximately 27–47%). This pattern suggests that the proposed oxidative stress panels may be more suitable as screening or supportive tools rather than definitive diagnostic markers. Among them, the MDA+TOS combination demonstrated the best overall performance, although its clinical applicability requires validation. Importantly, due to the lack of internal validation, these results should be interpreted cautiously, as model performance may be overestimated. Furthermore, oxidative stress markers were associated with metabolic parameters, including lipid profile, inflammatory marker, and body composition. Importantly, this analysis was conducted in a female-only cohort, addressing a gap in the literature despite the higher incidence of PTC in women (31). Previous studies linked oxidative stress to angioinvasion in PTC but lacked control groups and sex-specific analyses. Our findings support oxidative imbalance as a component of tumor aggressiveness, potentially influenced by hormonal and metabolic factors in women. Estrogen has been shown to promote PTC proliferation, migration, and invasion via the ERα/KRT19 pathway, highlighting the relevance of sex-specific approaches (8). Estrogen may modulate oxidative balance indirectly through metabolic effects, as adipose tissue serves both as a source of estrogen and a producer of pro-oxidant cytokines, potentially contributing to sex-related differences in tumor behavior (27, 28). In this study, associations between oxidative markers and body composition support the link between metabolic status and redox balance in PTC. OGG1 and MDA were elevated in PTC compared to controls, indicating tumor-associated oxidative stress. Higher levels in non-angioinvasive PTC may reflect more effective redox regulation and DNA repair, whereas lower levels in angioinvasive cases could indicate adaptation to chronic oxidative stress or exhaustion of repair mechanisms (32).

These findings highlight the dynamic relationship between oxidative stress and tumor progression, suggesting that the role of specific redox markers may differ depending on tumor aggressiveness (19–21, 33, 34). Moreover, associations between oxidative stress markers and thyroid-specific parameters suggest interactions between redox imbalance and tumor-related processes. In the angioinvasive PTC group, the inverse relationship between OGG1 and Tg may reflect fluctuations in oxidative DNA repair activity in relation to tumor burden following thyroidectomy, although this finding should be interpreted cautiously given known limitations of Tg assessment, including residual tissue and antibody interference. The positive correlation between TgAb and 3-NT suggests that enhanced nitrosative stress may be associated with a more immunogenic and aggressive tumor phenotype (18). Conversely, the inverse relationship between TgAb and MDA, despite their overall elevation, may reflect differential regulation of oxidative pathways, where lipid peroxidation is less dominant or alternative oxidative mechanisms, such as protein or DNA oxidation, are involved (35). This may also indicate that TgAb-positive patients exhibit stronger autoimmune control, potentially limiting lipid peroxidation in the tumor microenvironment (36). Clinically, this highlights the complex interplay between autoimmunity and oxidative damage in PTC and suggests that TgAb status does not uniformly reflect lipid peroxidation levels. These findings support a potential cross-talk between oxidative and immune pathways, where redox-related modifications may influence antigenicity and contribute to the autoimmune features of aggressive PTC (37). In the non-invasive PTC group, positive correlations between OGG1, MDA, TOS and lipid/inflammatory marker (CHOL, LDL, CRP) highlight the interplay between dyslipidemia, oxidative imbalance, and early tumor biology (38). In PTC, these processes may contribute to vascular invasion and tumor aggressiveness, consistent with the observed association between OGG1 and angioinvasion (18, 39). In the control group, physiological correlations of MDA, TOS, and TAS/TAC with metabolic and thyroid parameters support the biological validity of these markers. These findings indicate a dual role of oxidative stress markers as indicators of both tumor-related metabolic dysregulation and systemic redox status. Clinically, elevated OGG1 and MDA in the context of dyslipidemia or inflammation may support future risk stratification strategies, potentially identifying patients at higher risk of angioinvasion. Additionally, the inverse relationship between 8-OHdG and adiposity suggests a potential link between metabolic status and oxidative DNA damage, warranting further investigation. Our previous studies including both male and female patients demonstrated elevated oxidative stress in angioinvasive PTC, confirming oxidative imbalance as a core mechanism of tumor aggressiveness. Previous studies also indicate that oxidative stress markers may be transiently elevated shortly after treatment, particularly following RAI, and tend to stabilize over time. Therefore, the chosen postoperative time point prior to RAI was intended to reduce treatment-related variability (18, 19, 21, 22).

Associations between oxidative stress markers, lipid profile, inflammatory marker (CRP), and thyroid function parameters support the link between systemic redox imbalance and metabolic and inflammatory processes in PTC, consistent with previous studies (18–22, 40). Their persistence in this female-only cohort suggests that oxidative imbalance may be sex-specific following magnitude or pattern due to hormonal and metabolic influences. Notably, this study integrates peripheral oxidative stress biomarkers with DXA-derived body composition parameters, providing insight into the interaction between redox status and metabolic phenotype in relation to angioinvasive behavior. In the angioinvasive group, 8-OHdG showed consistent negative associations with adiposity measures, suggesting that greater fat mass may act as a redox buffer or, alternatively, that lower adiposity reflects a more catabolic state in aggressive tumors. Although not previously reported in PTC, similar findings in other malignancies suggest a potential protective role of adipose tissue in systemic oxidative stress regulation (17, 41). These findings suggest that hormonal and metabolic factors may jointly influence redox-related pathways involved in tumor behavior. Estrogen, adipose-derived cytokines, and altered lipid metabolism could jointly modulate ROS production, antioxidant responses, and DNA repair capacity, thereby shaping the tumor’s invasive phenotype (31, 42–44).

One limitation of this study is the relatively small sample size and the exclusive inclusion of female patients, which, while improving homogeneity, may limit generalizability, particularly for multivariable analyses. Therefore, logistic regression models were used in an exploratory manner and restricted to a limited number of predictors, and the findings should be considered hypothesis-generating and require validation in larger, independent cohorts. Menopausal status was not systematically assessed and may represent a potential confounding factor influencing hormonal, metabolic, and oxidative stress parameters. Oxidative stress markers were assessed only post-thyroidectomy, as angioinvasion can be determined only postoperatively, which precludes dynamic assessment across disease stages and limits interpretation of temporal changes. Although blood sampling was performed several months after surgery, residual postoperative effects cannot be completely excluded. However, all patients were evaluated under comparable conditions, including TSH-suppressive levothyroxine therapy prior to RAI, with no prior RAI exposure. In addition, patients with postoperative complications or active inflammatory conditions were excluded, and CRP levels did not indicate ongoing inflammation at the time of sampling, reducing the likelihood of confounding by these factors. Nevertheless, postoperative hormonal suppression and metabolic adaptation may still influence systemic redox balance and should be considered when interpreting the results. The absence of a surgical control group further limits direct assessment of surgery-related effects. Finally, the cross-sectional design does not allow evaluation of long-term prognostic value.

To our knowledge, this is the first study to comprehensively analyze associations between serum oxidative stress markers and detailed DXA-derived body composition in a homogeneous cohort of female PTC patients, linking systemic metabolic factors with markers of tumor aggressiveness. A simple two-marker panel (MDA+TOS) demonstrated promising discriminative performance (AUC 0.8324) for angioinvasion, suggesting potential as a non-invasive, low-cost adjunct to histopathological assessment. This combination may be biologically justified, as MDA reflects lipid peroxidation as a downstream consequence of oxidative damage, whereas TOS represents the overall systemic oxidant burden. Thus, their combination may provide a more comprehensive assessment of redox imbalance. In this study, this panel demonstrated the highest discriminative performance among the evaluated models; however, these findings should be interpreted cautiously given the exploratory design. If validated in larger prospective cohorts, these biomarkers may support future preoperative risk stratification. However, given the exploratory design, lack of adjustment for multiple clinical confounders, and cross-sectional postoperative setting, these findings should be interpreted as hypothesis-generating. Further prospective studies incorporating preoperative sampling (e.g., after FNAB) and integration with imaging and metabolic parameters are needed to assess their clinical utility and dynamic behavior.

## 5 Conclusion

This study demonstrates that selected oxidative stress markers, particularly MDA and OGG1, differ between PTC patients and healthy controls and are associated with angioinvasion in a female-only cohort. These findings support the role of systemic oxidative stress as a component of tumor aggressiveness. A simple two-marker panel (MDA+TOS) showed moderate discriminative performance, indicating its potential as a non-invasive adjunct to histopathological assessment in the postoperative setting.

The observed associations between oxidative stress, metabolic parameters, and body composition further suggest that systemic redox imbalance reflects the interaction between tumor biology and metabolic status. Given the exploratory design, these findings should be interpreted cautiously and require validation in larger, prospective studies, including preoperative assessment.

## Data Availability

The raw data supporting the conclusions of this article will be made available by the authors, without undue reservation.
